# Lower Circulating C1q/TNF-Related Protein-3 (CTRP3) Levels Are Associated with Obesity: A Cross-Sectional Study

**DOI:** 10.1371/journal.pone.0133955

**Published:** 2015-07-29

**Authors:** Risa M. Wolf, Kimberley E. Steele, Leigh A. Peterson, Thomas H. Magnuson, Michael A. Schweitzer, G. William Wong

**Affiliations:** 1 Department of Pediatrics, The Johns Hopkins University School of Medicine, Baltimore, Maryland, 21205, United States of America; 2 Department of Surgery, The Johns Hopkins Center for Bariatric Surgery, Baltimore, Maryland, 21205, United States of America; 3 Department of Physiology, The Johns Hopkins University School of Medicine, Baltimore, Maryland, 21205, United States of America; 4 The Center for Metabolism and Obesity Research The Johns Hopkins University School of Medicine, Baltimore, Maryland, 21205, United States of America; Northeast Ohio Medical University, UNITED STATES

## Abstract

**Purpose:**

C1q/TNF-related protein-3 (CTRP3) is a novel adipokine that lowers blood glucose levels, reduces liver triglyceride synthesis, and is protective against hepatic steatosis in diet-induced obese mouse models. We hypothesized that higher circulating serum levels of CTRP3 would be associated with a lean body mass index (BMI) and a more favorable metabolic profile in humans. The aim of this study was to investigate CTRP3 levels in lean individuals compared to obese individuals.

**Methods:**

This was a cross-sectional study of obese (n=44) and lean control patients (n=60). Fasting metabolic parameters were measured in all patients and serum CTRP3 levels were measured by ELISA.

**Results:**

BMI of the lean group was 21.9 ± 0.2 kg/m^2^ and obese group was 45.2 ± 1.1 kg/m^2^. We found significantly lower circulating levels of CTRP3 in obese individuals (405 ± 8.3 vs. 436± 6.7ng/mL, p=0.004) compared to the lean group. Serum CTRP3 levels were inversely correlated with BMI (p=0.001), and triglycerides (p<0.001), and significantly associated with gender (p<0.01), ethnicity (p=0.05), HDL-cholesterol (p<0.01), and adiponectin (p<0.01). We found BMI (p<0.01), gender (p<0.01), and ethnicity (p<0.05) to be significant predictors of CTRP3 levels when controlling for age in multiple regression analysis.

**Conclusions:**

CTRP3 is a beneficial adipokine whose circulating levels are significantly lower in obese individuals. Obesity causes dysregulation in adipokine production, including the down-regulation of CTRP3. Lower CTRP3 levels may contribute to the pathophysiology of metabolic disorders associated with obesity. Optimizing CTRP3 levels through novel therapies may improve obesity and its comorbidities.

## Introduction

Obesity has become a world-wide pandemic, and in the United States alone, it is estimated that two-thirds of adults are overweight or obese [1]. Obese individuals are at higher risk of obesity-associated comorbidities, including metabolic syndrome, type 2 diabetes, hyperlipidemia and heart disease [2,3].

Adipose tissue is an active endocrine organ that secretes adipokines, or fat-derived hormones, that function in maintaining metabolic homeostasis [4,5]. Obesity alters the function of these adipokines and contributes to metabolic dysregulation [4,5]. Some adipokines, such as adiponectin, are well studied, while the physiological function of many other adipokines remains incompletely understood. Adiponectin is a multifunctional, insulin-sensitizing adipokine known to regulate many aspects of glucose and lipid homeostasis [6,7]. Our laboratory has recently demonstrated that a novel family of secreted plasma proteins, the C1q/TNF-related proteins (CTRPs), similar to adiponectin, plays important roles in regulating glucose and lipid metabolism [8–18]. Our initial investigations of the novel adipokine CTRP3 (also known as cartonectin, cartducin, CORS-26) demonstrated that its circulating levels are lower in diet-induced obese mice [19]. Furthermore, we have shown that CTRP3 regulates gluconeogenesis and lipid metabolism in the liver [19,20]. Other investigators have determined that CTRP3 also has anti-inflammatory properties [21–24] and may be cardio-protective [25].

Because of its role in metabolism and obesity in rodent models, there has been recent investigation of CTRP3 in humans. Two studies investigating CTRP3 levels and its association with diabetes and metabolic syndrome reported conflicting results. One study showed elevated CTRP3 levels in patients with type 2 diabetes [26], while a more recent study reported lower levels of CTRP3 in newly-diagnosed patients with type 2 diabetes [27]. Women with Polycystic Ovarian Syndrome (PCOS) were found to have lower CTRP3 levels compared to their matched controls [28], and individuals with acute coronary syndrome or stable angina pectoris were also found to have decreased levels of CTRP-3 compared to control subjects [29].

In this study, our aim was to determine whether serum CTRP3 levels in humans were altered by obesity, and how CTRP3 levels are related to other metabolic parameters. To our knowledge, this is the first study in humans examining CTRP3 levels in the context of obesity.

## Research Design and Methods

### Study Design and Participants

This was a cross-sectional study conducted from October 2013 to October 2014 at the Johns Hopkins Hospital and the Johns Hopkins Bayview Medical Center. Obese patients were recruited from the Johns Hopkins Center for Bariatric Surgery. The inclusion criterion was eligibility for bariatric surgery, specifically body mass index (BMI) >35kg/m^2^. Patients with previous weight loss surgery were excluded. The obese cohort included 11 patients with type 2 Diabetes, 27 patients with hypertension, and 9 patients with hypercholesterolemia. Lean patients with a BMI ≤ 26 kg/m^2^ were recruited as controls at the Johns Hopkins Hospital. Lean patients with a history of diabetes or cardiovascular disease were excluded. Participants were verbally briefed about the study and signed written informed consent forms. All human studies were approved by the Johns Hopkins University School of Medicine Institutional Review Board.

### Clinical and Laboratory Measurements

Body mass index (BMI) was calculated as weight/height^2^ (kg/m^2^). All blood samples were obtained in the morning following an overnight fast. Blood samples were centrifuged at 3,200g for 7 min and serum was aliquoted and stored at -80°C for subsequent assays. Fasting blood glucose, aspartate aminotransferase (AST), alanine aminotransferase (ALT), cholesterol, triglycerides (TGL), high density lipoprotein cholesterol (HDL), low density lipoprotein cholesterol (LDL) and hemoglobin A1c (HbA1c) were performed by the Johns Hopkins Pathology Core Laboratory. Serum insulin levels were measured by ELISA (Millipore Human Insulin, Billerica, MA, USA). Insulin resistance was calculated with the homeostasis model assessment of insulin resistance (HOMA-IR) [30]. Human adiponectin and CTRP3 levels were measured by ELISA (AdipoGen, Incheon, Korea). Leptin was measured by Qauntikine ELISA (R&D Systems, Minneapolis, MN, USA).

Intra-assay coefficients of variation (CV) were 7.3 ± 1.0 (CTRP3), 3.1 ± 0.1 (Leptin), and 3.4 ±0.4 (Adiponectin). Inter-assay CVs were 5.8 ± 2.6 (CTRP3), 4.3 ±0.9 (Leptin), and 4.3 ±1.2 (Adiponectin).

### Statistical Analysis

Continuous variables were normally distributed, and are presented as mean +/- standard error of the mean (SEM), with ranges. Categorical variables are expressed as proportions (percentage). Student’s t-test was used to compare demographic and biochemical variables between the obese and lean groups. CTRP3 levels between genders was analyzed by two-tailed t-test, and CTRP3 levels among ethnicities were compared using a one-way ANOVA. Ethnicity was then categorized as White vs. non-White (including Black, Asian and Hispanic) for further analysis. Correlations between CTRP3 and continuous variables (BMI, LFTs, HbA1c, insulin, glucose, HOMA-IR and cholesterol levels) were analyzed using Pearson’s correlations and linear regression. Linear regression analysis was performed for each independent variable with CTRP3. If variables reached statistical significance on univariate analysis, they were included in multivariate analysis with CTRP3 as a dependent variable. A subset analysis was performed on obese patients with diabetes, hypertension and hypercholesterolemia. All statistical analyses were performed using STATA statistical software 11.0 (StataCorp LP, College Station, TX). A *p*-value of <0.05 was considered significant.

## Results

### Patient Demographics and Baseline Clinical Characteristics

Our cohort included 104 patients (27 men and 77 women) with a mean age of 38 ± 1.1 years (range 23–66). BMI of the lean group (n = 60) was 21.9 ± 0.2 kg/m^2^ (range 17.6–26) and the obese group (n = 44) was 45.2 ± 1.1 kg/m^2^ (range 35.6–68.6). The lean group included 41 (67%) Caucasian, 5 (8%) Black, 5 (8%) Hispanic, and 9 (15%) Asian individuals. The obese patient group included 29 (66%) Caucasians, 12 (27%) Blacks, and 3 (7%) Asians. Baseline metabolic parameters were within the normal range for all laboratory testing in the lean group. The baseline characteristics of all study subjects are summarized in Table 1. In the obese group, 11 had a diagnosis of type 2 diabetes, 27 had hypertension, and 9 had hypercholesterolemia. Renal function was normal in all of the obese patients, with an eGFR >60mL/min/1.73m^2^. The obese group showed a significantly higher mean BMI, fasting glucose, HbA1c, AST, and triglyceride levels when compared to the lean control group. HDL-cholesterol and adiponectin were significantly lower in the obese group compared to the lean group. We found significantly lower circulating CTRP3 levels in the obese group than the lean controls (405 ± 8.3 vs. 436± 6.7ng/mL, p = 0.004), and significantly higher levels of leptin in the obese group when compared to the lean group.

**Table 1:** Baseline Characteristics of Study Subjects. Data are means, with range. P-values calculated by two-tailed t-test.

	Control group	Obese group	p-value
	(n = 60)	(n = 44)	
Sex, female n(%)	40 (60)	38 (86)	
Age (years)	36.8 (23–59)	40.3 (23–66)	0.09
Body Mass Index (kg/m2)	21.9 (17.6–26)	45.2 (35.6–68.6)	<0.0001
AST (IU/L)	19.9 (12–47)	17.9 (7–42)	0.2
ALT (IU/L)	16.1 (8–42)	23.6 (7–81)	<0.001
Total cholesterol (mg/dL)	171.4 (120–258)	173.6 (127–239)	0.88
Triglycerides (mg/dL)	73.1 (39–165)	143.3 (40–651)	<0.0001
HDL cholesterol (mg/dL)	66.3 (30–93)	47.4 (29–79)	<0.0001
LDL cholesterol (mg/dL)	90.4 (55–151)	99 (46–151)	0.27
Glucose (mg/dL)	74.4 (56–106)	111.7 (75–259)	<0.0001
Insulin (uU/mL)	2.94 (0–13.4)	6.4 (1–17.9)	<0.0001
HOMA-IR	0.53 (0.2–6.12)	1.73 (0.2–5.0)	<0.0001
Hemoglobin A1C (%)	4.9 (4.5–5.8)	6.4 (4.7–10.4)	<0.0001
Adiponectin (ng/mL)	12056 (4622–32077)	6308 (3304–12575)	<0.0001
CTRP3 (ng/mL)	430.9 (350.7–634.7)	405.1 (301.5–560.9)	0.004
Leptin (pg/mL)	8343 (658.6–52585.2)	67854 (1825–163543)	<0.0001

### Association between CTRP3 levels and metabolic parameters

**Fig 1:**
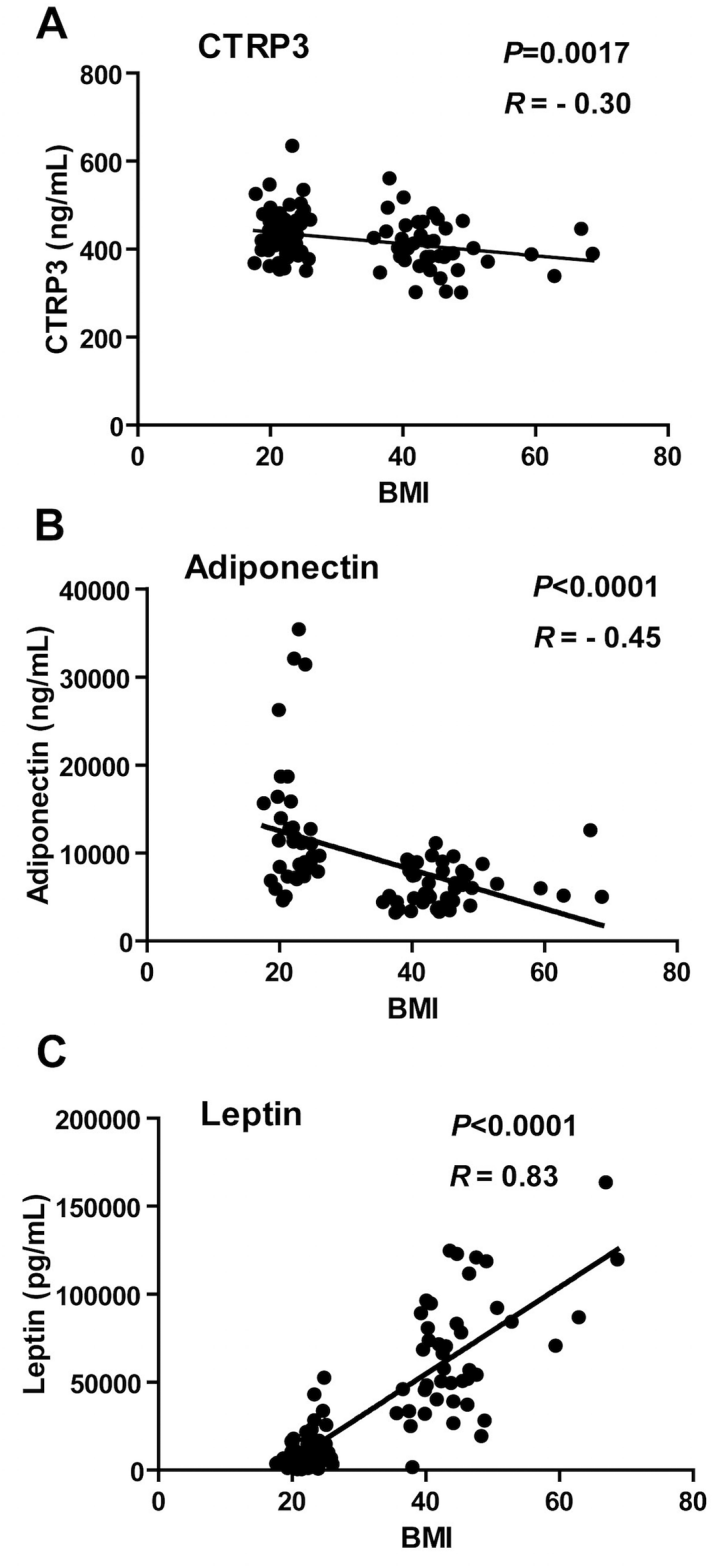
(A) and (B) CTRP3 is inversely related to BMI, analogous to adiponectin. (A) and (C) CTRP3 and Leptin have opposite relationships with BMI. Pearson’s r = correlation coefficient (p<0.05).

There was no difference in CTRP3 levels by age, but men had significantly lower CTRP3 levels compared to women (397.7 vs. 432 ng/mL, p<0.01). CTRP3 levels differed among ethnic groups (p = 0.05). When categorized as White vs. non-White (Black, Asian, and Hispanic), White patients had significantly lower mean CTRP3 levels (413.78 vs. 442.35 ng/mL, p = 0.01) compared to other ethnic groups. As shown in Table 2, in the lean controls, CTRP3 was correlated with leptin (p<0.01), and was borderline significant when correlated with HDL-cholesterol (p = 0.05). In obese patients, CTRP3 was inversely correlated with triglyceride levels (p<0.05). In the overall cohort, CTRP3 was inversely correlated with BMI (p = 0.001), triglycerides (p<0.001), and HOMA-IR (p = 0.05), and positively correlated with HDL-cholesterol (p<0.01), and adiponectin (p<0.01). CTRP3 was associated with gender (p<0.01) and ethnicity (p = 0.05). CTRP3 and adiponectin are both inversely correlated with BMI, whereas leptin has a positive relationship with BMI. (Fig 1)

**Table 2:** Association between CTRP3 levels and metabolic parameters. Pearson correlation was used for calculation of associations between variables.

	CTRP3					
	Lean		Obese		Combined	
	r	p	r	p	r	p
Sex^1^	--	**<0.0001**	--	0.38	--	**0.005**
Age	0.2	0.12	0.12	0.42	0.11	0.24
Ethnicity^2^	--	0.07	--	0.07	--	**0.05**
BMI	0.05	0.66	-0.21	0.15	-0.3	**0.0017**
AST	0.03	0.81	-0.004	0.97	0.04	0.62
ALT	0.02	0.86	0.04	0.79	-0.05	0.55
Total Cholesterol	0.004	0.97	-0.09	0.56	-0.02	0.79
Triglycerides	-0.05	0.64	-0.36	**0.02**	-0.33	**0.0007**
LDL- Cholesterol	-0.02	0.86	0.05	0.72	-0.02	0.79
HDL- Cholesterol	0.24	**0.05**	0.04	0.77	0.29	**0.003**
Fasting glucose	-0.05	0.7	-0.02	0.86	-0.15	0.12
Insulin	-0.05	0.67	-0.04	0.76	-0.17	0.07
Hemoglobin A1C	0.1	0.41	0.13	0.48	-0.09	0.38
HOMA-IR	-0.12	0.34	-0.009	0.95	-0.19	**0.05**
Adiponectin	0.22	0.18	0.07	0.63	0.29	**0.007**
Leptin	0.45	**0.0002**	-0.01	0.93	-0.14	0.15

^1^Two-tailed t-test was used to compare CTRP3 levels and gender.

^2^ANOVA was used to compare CTRP3 levels by ethnicity.

As shown in Table 3, univariate analysis revealed that gender, BMI, Ethnicity (defined as White vs. non-White), triglycerides, HDL-cholesterol, adiponectin and HOMA-IR were independently associated with CTRP3 levels. Of these, Ethnicity (defined as White vs. non-White), BMI, and triglycerides were inversely associated with CTRP3. Multiple regression analysis revealed that gender, ethnicity and BMI are significant predictors of CTRP3 levels when controlling for age. The metabolic parameters of triglycerides (β = -0.12, p = 0.09), HDL-cholesterol (β = 0.20, p = 0.51), adiponectin (β = 0.002, p = 0.07), and HOMA-IR (β = 4.6, p = 0.50) did not retain their significance as predictors of CTRP3 when controlling for age, gender, ethnicity and BMI.

**Table 3:** Univariate and multivariate analysis of the relationship between CTRP3 and metabolic parameters.

	Univariate Analysis		Multivariate Analysis	
	β	p-value	β	p-value
Sex	34	**0.005**	38.7	**<0.01**
Age(years)	0.55	0.25	0.54	0.2
Ethnicity*	-28.57	**0.013**	-24.2	**0.02**
BMI	-1.34	**0.002**	-1.6	**<0.01**
AST	0.41	0.62		
ALT	-0.3	0.55		
Total Cholesterol	-0.05	0.79		
Triglycerides	-0.244	**0.001**		
LDL- Cholesterol	-0.05	0.79		
HDL- Cholesterol	0.99	**0.003**		
Fasting glucose	-0.24	0.12		
Insulin	-2.6	0.07		
Hemoglobin A1C	-4.9	0.38		
HOMA-IR	-10.1	**0.05**		
Adiponectin	0.002	**0.008**		
Leptin	-0.0002	0.15		

*Ethnicity (white v non-white). B:unstandardized coefficient; R2 of the final multivariate model was 0.26 (p <0.01)

## Discussion

This is the first human study to report that circulating CTRP3 levels are significantly lower in obese versus non-obese subjects. Furthermore, BMI is a predictor of CTRP3 levels, even after controlling for potential confounders, including age, ethnicity and gender. This is also the first study that includes patients of multiple ethnicities, and we found lower levels of CTRP3 in Caucasians when compared to African American, Hispanic and Asian subjects. Women have higher CTRP3 levels than men, and gender is a significant predictor of CTRP3 levels in both univariate and multivariate regression models. Correlation studies showed that CTRP3 was positively associated with gender, HDL-cholesterol and adiponectin, and inversely associated with BMI, triglycerides, and leptin. Several of these associations persisted in a multivariate model, with gender, BMI, and ethnicity emerging as significant predictors of CTRP3 levels. BMI is a significant predictor of CTRP3 levels when controlling for age, gender and ethnicity.

Our laboratory first described CTRP3 as a novel adipokine that is decreased in diet-induced obese mice and inversely correlated with leptin levels [19]. This motivated our current study design and hypothesis that circulating CTRP3 levels would be lower in obese individuals. Similar to our rodent findings *in vivo* [19], we found that CTRP3 levels are significantly decreased in obese individuals, and are inversely correlated with leptin levels in obese humans. Yoo et al found no difference in CTRP3 levels between Korean patients with and without metabolic syndrome. They did identify an inverse correlation between CTRP3 and waist circumference, but not BMI [31].

Previous studies from our laboratory [19,20] and that of others suggest that CTRP3 is a beneficial metabolic hormone with anti-inflammatory properties, whose circulating levels are down-regulated in the pro-inflammatory obese state [21,32]. The expansion of visceral adipose tissue in obesity promotes the release of inflammatory cytokines, leading to a chronic low-grade inflammatory state which is linked to the comorbidities of type 2 diabetes and atherosclerosis [33]. CTRP3 is a secreted plasma protein structurally homologous to adiponectin [18]. While the widely-studied adiponectin has insulin-sensitizing, anti-inflammatory, and anti-atherogenic properties [4,6], the *in vivo* metabolic function of CTRP3 has only begun to be elucidated [19,20]. Circulating levels of adiponectin are known to be significantly reduced in obese and diabetic patients and further decreased in subjects with cardiovascular disease [34,35]. Our results show that both adiponectin and CTRP3 levels are significantly reduced in obese individuals relative to lean controls; in our cohort, CTRP3 levels are positively correlated with that of adiponectin. The aforementioned study by Yoo et al also showed a positive correlation between CTRP3 and adiponectin [31].

We previously demonstrated that recombinant CTRP3 administration lowers blood glucose in both normal and insulin resistant obese (*ob/ob*) mice, in part, by suppressing hepatic glucose output via activation of the protein kinase B/Akt signaling pathway [19]. We also showed that transgenic over-expression of CTRP3 in mice suppressed hepatic triglyceride synthesis and prevented the development of liver steatosis induced by high-fat feeding [20]. Based on our previous *in vivo* mouse model data, we would expect higher CTRP3 levels to correlate with a more favorable glucose and lipid profiles; and alternatively, patients with diabetes would have lower CTRP3 levels. In the first study by Choi et al examining CTRP3 levels in Korean patients with and without diabetes, the authors reported an unexpected elevation in CTRP3 levels in subjects with type 2 diabetes [26]. A subsequent follow up study by the same group, however, reported significant negative correlations of CTRP3 with metabolic risk factors, discordant from their previous findings [31]. The conflicting results were attributed to several possible causes, including small sample sizes, possible compensatory effect of CTRP3, and/or the medications used by the diabetes patients; the last point is particularly relevant as several glucose-lowering medications (e.g., GLP-1 receptor agonists and metformin) are now known to affect CTRP3 levels [28,36]. Recently, Ban et al assessed CTRP3 levels in newly diagnosed diabetics not on any glucose-lowering medications [27]. Their results showed that CTRP3 levels were significantly lower in individuals with type 2 Diabetes. Our cohort included 11 patients with type 2 Diabetes and 9 patients with impaired fasting blood glucose (>99 mg/dL). CTRP3 levels in the subgroup with diabetes and impaired fasting blood glucose was lower than those with normal glycemia (404.5 vs 428.1, p = 0.07), a trend that is consistent with the Ban et al study [27]. Comparison of patients on metformin (n = 11) to the entire obese cohort shows no difference in CTRP3 levels (401.1 vs 405.1, p = 0.83).

Our results further establish the relationship between CTRP3 and several metabolic parameters. Similar to findings in patients with metabolic syndrome and PCOS, we found serum triglyceride levels to be inversely correlated with CTRP3 levels [28,31]. Previous studies in patients with type 2 Diabetes and metabolic syndrome have observed an inverse correlation of fasting blood glucose with CTRP3 [27,31], and a study in women with PCOS found insulin to be an independent predictor of CTRP3 [28]. We have also observed that HDL-cholesterol levels are correlated with CTRP3. As metabolic parameters reach unhealthy levels, there develops an inverse relationship with CTRP3, further supporting that CTRP3 is a beneficial hormone that decreases in obesity, as shown in the present study, and in other pathophysiological conditions such as diabetes and cardiovascular disease [27,29].

One of the strengths of this study is that it is the first to investigate CTRP3 levels in a cohort that includes multiple ethnicities, and multivariate regression analysis found ethnicity to be a significant predictor of CTRP3 levels. This is an important finding, as significant health disparities exist between different ethnic groups, especially in relation to obesity, diabetes and cardiovascular disease [37,38]. Investigations of other adipokines and ethnicity have shown that adiponectin levels are lower, and leptin levels are higher in African American women compared to Caucasian women [39,40]. Consistent with these studies, we also observed significantly higher levels of leptin in our Black and Hispanic patients compared to White (p<0.001), but we did not find any differences in adiponectin levels by ethnicity. Identifying novel factors that account for these differences may impact our future investigation and treatments to bridge the disparity among different populations. Furthermore, we also found a significant correlation between gender and CTRP3 levels, with women exhibiting higher CTRP3 levels than men. One previous study in Korean individuals with metabolic syndrome also found this gender difference [26], and we replicate the findings in a cohort with multiple ethnicities.

A limitation of our study is that some patients in the lean cohort (24/60) were on medications (including multivitamins, oral contraceptive, or levothyroxine) at the time of enrollment. Comparison of their CTRP3 levels with other lean subjects without medications showed no differences in CTRP3 levels, and all were within the overall range of CTRP3 values. Additionally, some of the obese patients were taking medications for diabetes, hypertension and hypercholesterolemia, which might unknowingly affect their CTRP3 levels. Although we have a diverse patient population, our individual ethnic cohorts are small and thus our results should be interpreted with caution. Nonetheless, our findings highlight a significant relationship between CTRP3 levels and ethnicity.

In conclusion, our study demonstrates that CTRP3 levels are reduced in human obesity. The obese state alters normal metabolic homeostasis and leads to dysregulation of adipokine production and function. CTRP3, or other adipokines, may be promising targets for pharmacologic agents to treat obesity and its associated comorbidities. Future investigations should involve larger studies with patients of multiple ethnicities, to further elucidate the interesting relationship between serum CTRP3 levels and ethnicity.
